# Normal values of high-resolution transmural perfusion distribution metrics for automated quantitative pixel-wise myocardial perfusion cardiovascular magnetic resonance

**DOI:** 10.1016/j.jocmr.2025.101927

**Published:** 2025-06-19

**Authors:** Christel H. Kamani, Louise Brown, Thomas Anderton, Raluca Tomoaia, Chin Soo, Gaurav S. Gulsin, David A. Broadbent, Jian L. Yeo, Alice L. Wood, Christopher E.D. Saunderson, Ioannis Botis, Arka Das, Nicholas Jex, Amrit Chowdhary, Sharmaine Thirunavukarasu, Noor Sharrack, Peter P. Swoboda, Hui Xue, John P. Greenwood, David Adlam, Eylem Levelt, Gerry P. McCann, Peter Kellman, Sven Plein

**Affiliations:** a Department of Biomedical Imaging Science, Leeds Institute of Cardiovascular and Metabolic Medicine, University of Leeds, Clarendon Way, Leeds, UK; b Department of Cardiology, Lausanne University Teaching Hospital (CHUV), Lausanne, Switzerland; c Department of Nuclear Medicine and Molecular Imaging, Lausanne University Teaching Hospital (CHUV), Lausanne, Switzerland; d “Iuliu Hatieganu” University of Medicine and Pharmacy, Cluj-Napoca, Romania; e Department of Cardiovascular Sciences, University of Leicester and Cardiovascular Theme, NIHR Leicester Biomedical Research Centre, Glenfield Hospital, Leicester, UK; f Medical Physics and Engineering, Leeds Teaching Hospitals NHS Trust, Leeds, UK; g National Heart, Lung, and Blood Institute, National Institutes of Health, DHHS, Bethesda, Maryland, USA

**Keywords:** Normal values, Myocardial perfusion, Endocardial and epicardial layer, Cardiovascular magnetic resonance

## Abstract

**Background:**

The myocardial blood flow (MBF) transmural distribution between the subendocardial (ENDO) and subepicardial (EPI) layers under resting and hyperemic conditions can aid in the diagnosis of several forms of heart disease. Recently proposed automated in-line myocardial perfusion cardiovascular magnetic resonance (CMR) allows pixel-wise quantification of ENDO- and EPI-MBF, but normal values for these parameters are lacking. We therefore aimed to establish normal values for transmural distribution of MBF in a healthy population.

**Methods:**

138 healthy participants from two centers underwent adenosine stress and rest myocardial perfusion CMR. Global and myocardial slice-specific stress/rest ENDO- and EPI-MBF values were derived using pixel-wise in-line automatic post-processing, and transmural perfusion metrics (ENDO and EPI myocardial perfusion reserve [MPR_ENDO_, MPR_EPI_]; stress and rest ENDO-to-EPI gradient [sGRAD and rGRAD]) were computed using the Gadgetron software.

**Results:**

The study cohort comprised 84 males and 54 females (mean age: 50 ± 36) with no cardiovascular disease or risk factors. In the entire cohort, MPR_ENDO_ (3.3 ± 1.2) was significantly lower (p<0.001) than MPR_EPI_ (3.9 ± 1.2). sGRAD (0.98 ± 0.09) was significantly lower (p<0.001) than rGRAD (1.11 ± 0.07). “While there were no sex-specific differences in the majority of these metrics, all correlated inversely with increasing age. We propose specific values for each slice. These are conditional to the pulse sequence, acquisition timing and analysis method used in this work, as mean ± SD values at the basal, mid and apical level for MPR_ENDO_ (3.7 ± 1.1, 3.3 ± 0.9, 3.6 ± 1.0), MPR_EPI_ (4.0 ± 1.1, 3.9 ± 1.1, 4.0 ± 1.1), sGRAD (1.00 ± 0.13, 0.92 ± 0.09, 1.06 ± 0.18) and rGRAD (1.10 ± 0.09, 1.09 ± 0.07, 1.18 ± 0.11).

**Conclusion:**

Normal global and myocardial slice-specific values of MPR_ENDO_, MPR_EPI_, sGRAD and rGRAD using in-line automated MBF quantification from first pass myocardial perfusion CMR are presented. While there were no sex-specific differences in any of these metrics, all correlated inversely with increasing age. Understanding the MBF dynamics of the myocardial layers in healthy subjects will help to characterize MBF alterations in patients with coronary artery disease or microvascular dysfunction.

## Graphical abstract

**Figure:**
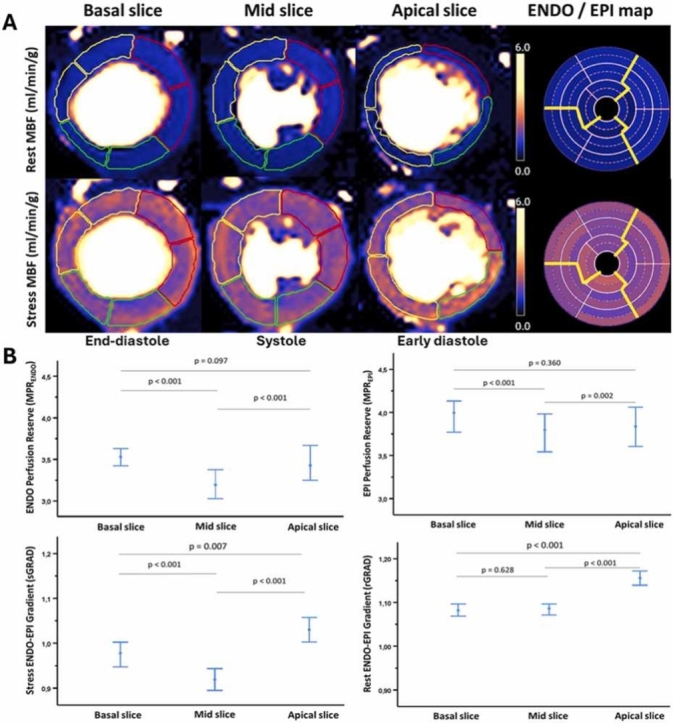
Automated myocardial delineation (A) and Myocardial slice-specific normal reference ranges for MPR_ENDO_, MPR_EPI_, sGRAD and rGRAD (B). Myocardial slice-specific normal reference ranges for MPR_ENDO_, MPR_EPI_, sGRAD and rGRAD, expressed as median values and 95% confidence interval. Myocardial slices are acquired in sequence during the R-R interval, resulting in each level representing a distinct cardiac phase: the basal slice aligns with end-diastole, the mid-ventricular slice with systole, and the apical slice with early diastole. *MPR*_*ENDO*_ Myocardial perfusion reserve in the endocardial layer, *MPR_EPI_* Myocardial perfusion reserve in the epicardial layer, *sGRAD* hyperemic endocardial to epicardial gradient, *rGRAD* rest endocardial to epicardial gradient

## 1. Introduction

Assessing the perfusion dynamics of subendocardial and subepicardial myocardial blood flow (MBF) may aid in the diagnosis of patients with coronary artery disease (CAD) and microvascular disease (MVD) [1]. Cardiovascular magnetic resonance (CMR) myocardial perfusion imaging is uniquely suited to quantify MBF in separate myocardial layers given its high in-plane spatial resolution. Previous studies have reported on the use of the transmural perfusion gradient, derived from myocardial perfusion CMR data to visualize and quantify the amplitude, extent and persistence of variations in endocardial (ENDO) to epicardial (EPI) blood flow [2]. The method demonstrated high accuracy for the identification of microvascular disease [3] as well as for the assessment of hemodynamically significant CAD as defined by fractional flow reserve (FFR) [4], [5]. Since then, important progress has been made in the field of quantitative myocardial perfusion CMR with the emergence of automated in-line methods for high-resolution pixel-wise estimation of MBF [6], [7]. Pixel-wise quantitative myocardial perfusion correlates well with quantitative myocardial perfusion positron emission tomography/computed tomography (PET/CT) [8] and has demonstrated high diagnostic accuracy and prognostic value in patients with suspected CAD [9], [10]. The method has also been shown to have higher diagnostic accuracy for the detection of multivessel CAD than visual assessment [11].

An important prerequisite for the wider use of layer-specific MBF values is access to normative data from a reference population that establishes the baseline distribution of transmural perfusion values. While normal values for transmural perfusion gradients have been presented for quantitative PET/CT perfusion [12], they are not currently available for CMR [13]. Given the higher spatial resolution of CMR compared with PET as well as the difference in the MBF assessment process, normal values may not be directly translatable between the two modalities. We therefore aimed to establish specific normal values for ENDO/EPI perfusion metrics derived from pixel-wise automated quantitative myocardial perfusion CMR in a large representative healthy population.

## 2. Methods

### 2.1. Study population

The study population was derived from a previously described cohort of 151 healthy volunteers with no major cardiovascular risk factors or history of cardiovascular disease, prospectively recruited from two cardiac tertiary centers (Leeds Teaching Hospitals Trust, Leeds, UK and University Hospitals of Leicester NHS Trust, Leicester, UK) [14]. Study exclusion criteria were a) known cardiovascular risk factors such as arterial hypertension, hypercholesterolemia, diabetes mellitus, smoking, b) previous documented CAD or revascularization, c) stress inducible perfusion defect on CMR, d) contraindication to adenosine, e) contraindication to gadolinium-based contrast agents or MRI and f) evidence of myocardial late gadolinium enhancement (LGE) on CMR. The study protocol was approved by institutional research ethics committees and complied with the Declaration of Helsinki. All subjects gave written informed consent for their participation.

### 2.2. Study protocol

This analysis used data that were acquired as part of a previously published study [14], [15]. Each participant underwent CMR on a 3 Tesla system (Prisma [Leeds] or Skyra [Leicester], Siemens Healthcare, Erlangen, Germany). The CMR protocol included cine imaging in multiple planes, adenosine stress and rest perfusion and LGE. Patients were instructed to avoid caffeine-containing food or beverages for 24 h prior to the scan.

Perfusion images were acquired with a dual sequence method as previously reported [7]. The arterial input function was acquired with a dual-echo acquisition (allowing for T2*-related signal loss correction). The myocardial tissue response was acquired with a saturation recovery pulse sequence with fast low-angle shot (FLASH) readout for [3-fold] acceleration using temporal generalized autocalibrating partial parallel acquisition (typical pulse sequence parameters: Flip angle = 14°; TE = 1 ms; TR = 146 ms, trigger delay/saturation recovery delay time [TD] = 72 ms, acquired voxel-size = 2.70 × 2.08 × 8.00 mm^3^). Linear phase encoding order was used with the truncated lines of k-space in latter half. Total imaging duration per slice including saturation recovery preparation and delay was 143 ms per slice. This allowed acquisition of the AIF and three myocardial slices at every RR interval up to a heart rate of 120bpm. The AIF was acquired in a single slice positioned in the basal left ventricle. The three myocardial slices were acquired at the basal, mid and apical LV planned using the “3 of 5” method. Data were acquired sequentially at each RR interval with a trigger delay from the R wave of 2.8 ms in the order; AIF slice followed by myocardial base, mid, and apical slices. As a result, allowing for variations due to heart rate and contractile function between individuals, the mid LV slice was generally acquired in the most systolic cardiac phase (approximately 300 ms from the R wave), while basal and apical slices were acquired in more diastolic phases. In the first three heartbeats, proton density–weighted images without saturation preparation were acquired to allow for baseline correction [7], [16].

Stress acquisitions: stress images were acquired during intravenous adenosine infusion (140 μg/kg/min for a minimum of 3 min). The dose was increased to a maximum of 210 μg/kg/min after 2 min if there was insufficient symptomatic or hemodynamic response, defined as a heart rate (HR) increase of less than 10 bpm. Hemodynamic parameters including blood pressure (BP), heart rate (HR), electrocardiogram as well as clinical symptoms (flushing, dyspnea, chest pain) were documented throughout the stress acquisition. At peak stress, a bolus of 0.05 to 0.075 mmol/kg gadolinium-based contrast agent (Gadovist, Bayer Healthcare, Leverkusen, Germany or Dotarem, Guerbet, Villepinte, France) was administered intravenously (antecubital fossa) using a power injector (MedRad ®, Bayer Healthcare) at a rate of 3–5 mL/sec followed by a 30 mL flush of normal saline at the same injection rate.

Rest acquisition: Following normalization of the heart rate to baseline, a cine data set covering the heart in short-axis orientation was acquired, followed by rest perfusion imaging (at least 15 min after stress) using the same acquisition protocol and contrast agent administration as for stress acquisition and LGE images in multiple planes.

### 2.3. In-line processing and quantitative analysis of dynamic perfusion data

**Fig. 1:**
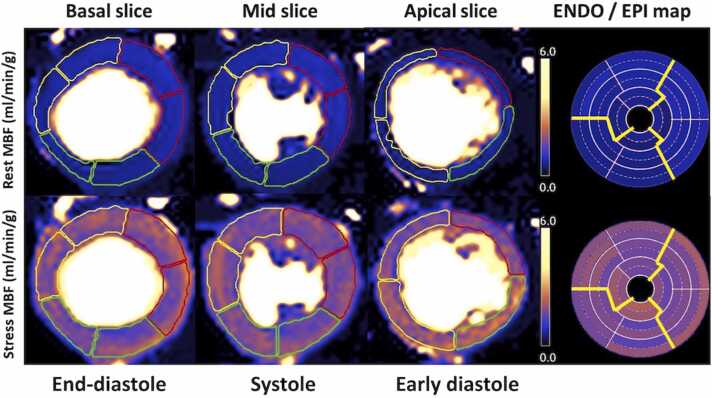
Representation of the rest and stress perfusion maps at basal, mid and apical slices, in mL/min/g. Mean segmental MBF values were averaged to obtain regional and global MBF values. Regional pixel-wise perfusion maps were delineated based on the 16-AHA standard nomenclature (yellow lines) [18]. Endo- and epi-myocardial layers were delineated using the ENDO and EPI border as well as a midventricular line [19]. Due to the sequential acquisition of the myocardial slices across the R-R interval (basal-mid-apical), each slice is acquired in a different cardiac phase (Basal slice—end-diastole/Mid slice—systole/Apical slice—early diastole). *MBF* Myocardial blood flow, *AHA* American Heart Association, *ENDO* endocardial, *EPI* epicardial

Post-processing of the acquired dynamic myocardial perfusion data used the Gadgetron software framework for in-line automatic reconstruction and post-processing as previously described [7], [14], [15]. The incidence of dark rim artifacts is minimized in our protocol design by using a short imaging duration (approximately 70 ms) [7]. Furthermore, raw filtering was applied to reduce edge ringing (Gibbs ringing) and to decrease measurement contamination caused by fat [17]. MBF at stress (sMBF) and rest (rMBF) were automatically derived and presented in mL/min/g on a pixel-wise map as well as for 16 myocardial segments according to the American Heart Association (AHA) classification [18]. The endo- and epicardial layers were automatically delineated from the endocardial and epicardial border as well as a midventricular line [19]. To delineate the midventricular line, we first determine the center of the LV blood pool and then compute rays at 1-degree increment. Then, the MBF map was interpolated to a spatial resolution of 1 mm^2^. Finally, the midventricular line was automatically positioned equidistantly between the endocardial and epicardial contours on the interpolated map (see Fig. 1). Perfusion maps were quality-controlled by a Level-3 CMR reader and any data with significant image artifact or other errors were excluded from final analysis.

Segmental sMBF and rMBF were automatically obtained as an average of all pixel values within each of the 16 segments. Regional sMBF and rMBF for the left anterior descending (LAD), circumflex (LCX) and right coronary artery (RCA) were automatically obtained as an average of all pixel values in the respective coronary territories as defined in the AHA classification. Global LV, regional sMBF and rMBF and MPR were also obtained separately for the endocardial and the epicardial layers. Stress and rest endocardial to epicardial perfusion gradients (sGRAD and rGRAD), were assessed as the ratio of sMBF_ENDO_ to sMBF_EPI_ and rMBF_ENDO_ to rMBF_EPI_, respectively, at global LV and regional level.

Rate pressure product (RPP) was obtained from the product of HR and systolic BP. RPP corrected rMBF (rMBF_c_) at global LV level was obtained from the ratio of each individual RPP value to 10,000 [20]. RPP corrected MPR (MPR_c_) was obtained as the ratio of sMBF to rMBF_c_. Normal values at LV level were proposed as mean ± 1 SD [95% CI].

Slice-specific values of the transmural gradients were obtained as the mean values of the corresponding LV basal, mid, and apical segments.

### 2.4. Statistical analysis

Normality was assessed using the Shapiro–Wilk test. Continuous variables were expressed as mean values ± standard deviation (SD) by normal distribution or median ± 25–75% interquartile range (IQR) when distribution was not normal. Comparison between groups was performed using related Friedman’s two way analysis of variance, Wilcoxon test and Mann-Whitney U test for non-normally distributed variables respectively one way analysis of variance (ANOVA) test, independent T-test and paired-simple T-test for normally distributed variables. Correlation was assessed using Spearman correlation coefficient for non-normally distributed variables and Pearson correlation coefficient for normally distributed variables. As proposed by previous publications, normal ranges were defined as the 95% cohort range [14], [21]. A p value <0.05 was considered statistically significant. Statistical analyses were performed in SPSS (version 29.0.0.1, Statistical Package for the Social Sciences, International Business Machines, Inc., Armonk, New York).

## 3. Results

From the initial cohort of 151 healthy volunteers prospectively included in the study, data from 13 subjects were excluded from the current analysis (1 participant due to arrythmia-related artifact and 12 participants due to missing stress or rest acquisition sequences). The final cohort of 138 patients included 84 males and 54 females, aged between 19 and 79 y (median 50 ± 35 IQR). Around 96 of these patients were recruited from Leeds and 42 from Leicester. In 18 patients, 0.075 mmol/kg gadolinium was administered per perfusion acquisition, while the remainder received 0.05 mmol/kg.

Using age- and sex-matched samples, there was no significant difference between MPR_ENDO_, MPR_EPI_,and sGRAD either between the two centers (n = 26); or when using 0.05 and 0.075 mmol/kg of contrast agent bolus (n = 20). rGRAD measurements were significantly higher in data from Leeds or when using 0.05 mmol/kg of contrast agent but these differences were no longer significant when rMBF values were corrected for RPP (Table 1, Table 2 of the supplementary material).

**Table 1:** Demographics and hemodynamic characteristics.

Parameters	All (n = 138)	Female (n = 54)	Male (n = 84)	p-value (F:M)
Age (y)	50 ± 36	33 ± 34	52 ± 34	0.175
Rest HR (bpm)	62 ± 15	65 ± 9	63 ± 9	0.144
Rest BP (mmHg)	118 ± 26	112 ± 26	120 ± 25	0.046
Rest RPP (mmHg × bpm)	7500 ± 2278	7773 ± 1892	7747 ± 1673	0.937
Stress HR (bpm)	89 ± 15	95 ± 15	86 ± 14	<0.001
Stress BP (mmHg)	120 ± 25	116 ± 22	125 ± 17	0.063
Stress RPP (mmHg x bpm)	10660 ± 3291	11186 ± 2992	10739 ± 2370	0.249
HR change (bpm)	26 ± 12	29 ± 17	23 ± 10	0.002
BP change (mmHg)	1 ± 9	1 ± 10	1 ± 10	0.825
RPP change (mmHg x bpm)	3093 ± 2065	3436 ± 1918	2992 ± 2380	0.083

Values are given as median (±IQR) or mean (±SD). The p-value indicates the significance of differences between sex. *BP* systolic blood pressure, *bpm* beat per minute, *HR* heart rate, *mmHg* millimeter of mercury, *RPP* rate pressure product, *IQR* interquartile range; *SD* standard devia

**Table 2:** Global and regional MBF values.

Parameters	All (n = 138)	Female (n = 54)	Male (n = 84)	p-value (F:M)
rMBF	0.60 ± 0.17	0.68 ± 0.13	0.56 ± 0.17	< 0.001
RPP-corrected rMBF	0.8 ± 0.3	0.92 ± 0.22	0.74 ± 0.23	< 0.001
sMBF	2.23 ± 0.53	2.41 ± 0.47	2.12 ± 0.53	0.001
MPR	3.64 ± 1.18	3.67 ± 0.99	3.79 ± 1.00	0.522
rMBF_LAD_	0.64 ± 0.19	0.74 ± 0.14	0.60 ± 0.17	< 0.001
rMBF_LCX_	0.58 ± 0.16	0.66 ± 0.14	0.54 ± 0.16	< 0.001
rMBF_RCA_	0.56 ± 0.20	0.64 ± 0.12	0.55 ± 0.11	<0.001
sMBF_LAD_	2.34 ± 0.56	2.53 ± 0.50	2.22 ± 0.57	0.001
sMBF_LCX_	2.23 ± 0.56	2.42 ± 0.50	2.10 ± 0.56	< 0.001
sMBF_RCA_	2.09 ± 0.49	2.23 ± 0.47	2.00 ± 0.49	0.008
MPR_LAD_	3.44 ± 1.09	3.33 ± 1.16	3.68 ± 0.96	0.270
MPR_LCX_	3.95 ± 1.68	3.91 ± 1.15	3.98 ± 1.18	0.749
MPR_RCA_	3.58 ± 1.09	3.49 ± 1.11	3.72 ± 0.98	0.320

Values are given as median (±IQR) or mean (±SD). The p-value indicates the significance of differences between sex. MBF values are given in mL/min/g myocardium. *MBF* myocardial blood flow, *rMBF* rest myocardial blood flow, *sMBF* stress myocardial blood flow, *MPR* myocardial perfusion reserve, *rMBF_LAD_* myocardial perfusion at rest in the LAD territory, *rMBF_LCX_* myocardial perfusion at rest in the LCX territory, *rMBF_RCA_* myocardial perfusion at rest in the RCA territory, *sMBF_LAD_* myocardial perfusion at stress in the LAD territory, *sMBF_LCX_* myocardial perfusion at stress in the LCX territory, *sMBF_RCA_* myocardial perfusion at stress in the RCA territory, *MPR_LAD_* myocardial perfusion reserve in the LAD territory, *MPR_LCX_* myocardial perfusion reserve in the LCX territory, *MPR_RCA_* myocardial perfusion reserve in the RCA territory, *IQR* interquartile range, *SD* standard deviation

## 4. Baseline characteristics

Table 1 gives the demographic and hemodynamic characteristics of the entire cohort, and separately for female and male participants. Stress HR (p<0.001) and HR change between rest and stress acquisitions (p = 0.002) were significantly higher in female than male participants, while rest BP (p = 0.046) was significantly higher in male than female participants. There were no other sex-specific differences.

## 5. Global and regional MBF

Table 2 shows the global and regional MBF parameters for the entire cohort, and separately for female and male participants. Global stress (2.41 ± 0.47 vs 2.12 ± 0.53 mL/g/min p = 0.001) and rest (0.68 ± 0.13 vs 0.56 ± 0.17 mL/g/min p<0.001) and all regional MBF values were significantly higher in female than male participants. There were no significant differences between female and male participants for global and regional MPR values.

## 6. Endo- and epicardial MPR

### 6.1. Global and regional MPR_ENDO_ and MPR_EPI_

Table 3 gives the global and endocardial and epicardial values of sMBF, rMBF and MPR for the entire cohort, and separately for female and male participants. There were no significant global sex-specific differences in the MPR_ENDO_ and MPR_EPI_ values.

**Table 3:** Global endo-epicardial MBF and MPR.

Parameters	All n = 138	Female n = 54	Male n = 84	p-value (F:M)
*LV*				
rMBF_ENDO_	0.63 ± 0.16	0.71 ± 0.13	0.59 ± 0.16	<0.001
rMBF_EPI_	0.57 ± 0.17	0.64 ± 0.12	0.54 ± 0.14	<0.001
sMBF_ENDO_	2.20 ± 0.52	2.39 ± 0.50	2.09 ± 0.50	<0.001
sMBF_EPI_	2.26 ± 0.55	2.39 ± 0.50	2.17 ± 0.56	0.008
MPR_ENDO_	3.3 ± 1.2	3.5 ± 1.0	3.5 ± 0.9	0.816
MPR_EPI_	3.9 ± 1.2	3.9 ± 1.1	4.0 ± 1.1	0.399

Values are given as median (±IQR) or mean (±SD). The p-value indicates the significance of differences between sex. *MBF* myocardial blood flow, *rMBF_ENDO_* Myocardial perfusion at rest in the endocardial layer, *rMBF_EPI_* Myocardial perfusion at rest in the epicardial layer, *sMBF_ENDO_* Myocardial perfusion at stress in the endocardial layer, *sMBF_EPI_* Myocardial perfusion at stress in the epicardial layer, *MPR_ENDO_* Myocardial perfusion reserve in the endocardial layer, *MPR_EPI_* Myocardial perfusion reserve in the epicardial layer, *IQR* interquartile range, *SD* standard deviation

rMBF_ENDO_ was significantly higher than rMBF_EPI_ (0.63 ± 0.16 vs 0.57 ± 0.17; p<0.001), in contrary to sMBF_ENDO_ which was significantly lower than sMBF_EPI_ (2.20 ± 0.52 vs 2.26 ± 0.55; p = 0.016).

Supplementary Table 3 gives regional endocardial and epicardial values of sMBF, rMBF and MPR for all participants and separately for female and male participants. Most ENDO and EPI stress and rest MBF values were higher in the LAD territory and lower in the RCA territory. There were no significant global sex-specific differences in the MPR_ENDO_ and MPR_EPI_ values. In the entire cohort, there was no significant difference between LV MPR_ENDO_ and MPR_ENDO-LAD_ (p = 0.153), MPR_ENDO-LCX_ (p = 0.273) or MPR_ENDO-RCA_ (p = 0.886). However, there was a significant difference between MPR_EPI_ and MPR_EPI-LAD_ (p = 0.001), MPR_EPI-LCX_ (p<0.001) and MPR_EPI-RCA_ (p = 0.026). MPR_ENDO_ was significantly lower than MPR_EPI_ (p<0.001).

### 6.2. Age-specific MPR_ENDO_ and MPR_EPI_

**Fig. 2:**
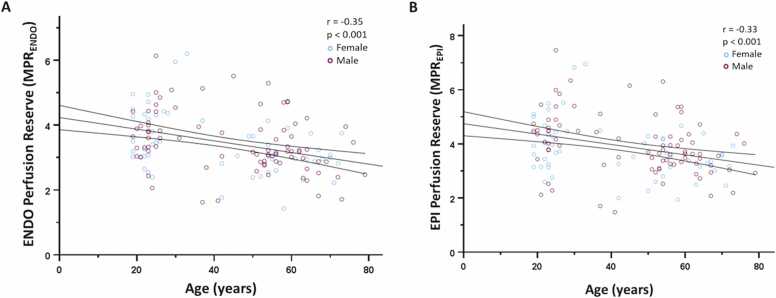
Correlation between (A) MPR_ENDO_ and age; (B) MPR_EPI_ and age. There is a significant inverse correlation between MPR_ENDO_ as well as MPR_EPI_ with increased age. *MPR*_*ENDO*_ myocardial perfusion reserve in the endocardial, *MPR*_*EPI*_ myocardial perfusion reserve in the epicardial

Fig. 2 A and B show a significant inverse correlation between MPR_ENDO_ (r = −0.35, p<0.001) and MPR_EPI_ (r = −0.33, p<0.001) values with increasing age. Interestingly, sMBF_ENDO_ (r = −0.467, p<0.001) and sMBF_EPI_ (r = −0.339, p<0.001) showed a significant inverse correlation with increasing age, while rMBF_ENDO_ (r = −0.094, p = 0.271) and rMBF_EPI_ (r = −0.035, p = 0.687) did not. Moreover, the age-related inverse correlation of MPR_ENDO_ and MPR_EPI_ with increasing age was no longer observed after RPP-adjustment of rMBF_ENDO_ (r = −0.107; p = 0.232) and rMBF_EPI_ (r = −0.061, p = 0.495).

### 6.3. Slice-specific MPR_ENDO_ and MPR_EPI_

Table 4 gives the slice-specific MPR_ENDO_ and MPR_EPI_ values for the entire cohort, and separately for female and male participants. There was no significant gender difference of MPR_ENDO_ and MPR_EPI_ values of the basal, mid and apical slices.

**Table 4:** Myocardial slice-specific MPR_ENDO_ and MPR_EPI_.

Parameters	All (n = 138)	Female (n = 54)	Male (n = 84)	p-value (F:M)
MPR_ENDO_				
Basal slice	3.5 ± 1.3	3.6 ± 1.1	3.6 ± 1.0	0.585
Mid slice	3.2 ± 1.1	3.18 ± 0.9	3.3 ± 0.8	0.366
Apical slice	3.4 ± 1.3	3.5 ± 1.0	3.6 ± 0.9	0.432
MPR_EPI_				
Basal slice	4.0 ± 1.3	3.9 ± 1.1	4.1 ± 1.1	0.449
Mid slice	3.8 ± 1.3	3.8 ± 1.1	3.9 ± 1.1	0.406
Apical slice	3.9 ± 1.4	3.9 ± 1.3	4.1 ± 1.1	0.424

Values are given as median (±IQR) or mean (±SD). The p-value indicates the significance of differences between sex. *MPR_ENDO_* Myocardial perfusion reserve in the endocardial layer, *MPR_EPI_* Myocardial perfusion reserve in the epicardial layer, *IQR* interquartile range, *SD* standard deviation

In the entire cohort, mid slice MPR_ENDO_ was significantly lower than basal MPR_ENDO_ (3.3 ± 1.1 vs. 3.5 ± 1.3; p<0.001) and apical MPR_ENDO_ (3.3 ± 1.1 vs. 3.6 ± 0.9; p<0.001). There was no significant difference between basal and apical slice MPR_ENDO_ (3.5 ± 1.3 vs. 3.6 ± 0.9; p = 0.097).

Mid slice MPR_EPI_ was significantly lower in comparison to basal MPR_EPI_ (3.8 ± 1.3 vs. 4.0 ± 1.3; p<0.001) and apical MPR_EPI_ (3.8 ± 1.3 vs. 3.9 ± 1.4; p = 0.002). There was no significant difference between basal and apical slice MPR_EPI_ (4.0 ± 1.3 vs. 3.9 ± 1.4; p = 0.360).

Supplementary Table 4 gives slice-specific values of sMBF_ENDO_, sMBF_EPI_, rMBF_ENDO_ and rMBF_EPI_ for all participants and separately for female and male participants. Apical rMBF values are consistently lower than basal and mid values, with Bonferroni-adjusted p-values showing strong significance (<0.001). Similarly, sMBF exhibits significant variations across segments, with higher values in the basal region compared to the apical region. Sex-based analysis shows that women have higher rMBF and sMBF values than men, with statistically significant differences in most segments (p<0.001), except for apical epicardial sMBF (2.14 ± 0.49 vs 2.02 ± 0.58, p = 0.162).

## 7. rGRAD and sGRAD

### 7.1. Global and regional rGRAD and sGRAD

Table 5 gives the global and regional rest and stress endo- to epicardial gradient values for the entire cohort, and separately for female and male participants. In female participants, sGRAD_LCX_ and rGRAD_LCX_ were significantly higher in comparison to male participants.

**Table 5:** Global and regional ENDO-to-EPI gradient.

Parameters	All (n = 138)	Female (n = 54)	Male (n = 84)	p-value (F:M)
LV global				
sGRAD	0.98 ± 0.09	1.00 ± 0.08	0.97 ± 0.08	0.115
rGRAD	1.11 ± 0.07	1.11 ± 0.07	1.11 ± 0.42	0.550
Coronary territory				
sGRAD_LAD_	0.98 ± 0.09	1.00 ± 0.10	0.97 ± 0.08	0.175
sGRAD_LCX_	0.95 ± 0.16	1.00 ± 0.12	0.94 ± 0.10	0.016
sGRAD_RCA_	1.00 ± 0.11	1.02 ± 0.11	0.99 ± 0.10	0.086
rGRAD_LAD_	1.08 ± 0.07	1.07 ± 0.05	1.09 ± 0.05	0.441
rGRAD_LCX_	1.15 ± 0.12	1.17 ± 0.14	1.14 ± 0.11	0.012
rGRAD_RCA_	1.10 ± 0.07	1.09 ± 0.06	1.10 ± 0.06	0.555

Values are given as median (±IQR) or mean (±SD). The p-value indicates the significance of differences between sex. *ENDO* endocardial layer, *EPI* epicardial layer, *sGRAD* hyperemic endocardial to epicardial gradient, *rGRAD* rest endocardial to epicardial gradient, *sGRAD_LAD_* sGRAD in the LAD territory, *sGRAD_LCX_* sGRAD in the LCX territory, *sGRAD_RCA_* sGRAD in the RCA territory, *rGRAD_LAD_* rGRAD in the LAD territory, *rGRAD_LCX_* rGRAD in the LCX territory, *rGRAD_RCA_* rGRAD in the RCA territory, *IQR* interquartile range, *SD* standard deviation

In the entire cohort, there was no significant difference between global sGRAD and sGRAD_LAD_(p = 0.705). However, there was a significant difference between global sGRAD and sGRAD_LCX_ (p = 0.021) as well as sGRAD_RCA_ (p = 0.003). There was a significant difference between global rGRAD and rGRAD_LAD_ (p<0.001), rGRAD_LCX_ (p<0.001) and rGRAD_RCA_ (p = 0.035). Global sGRAD was significantly lower than global rGRAD (p<0.001).

### 7.2. Age-specific rGRAD and sGRAD

**Fig. 3:**
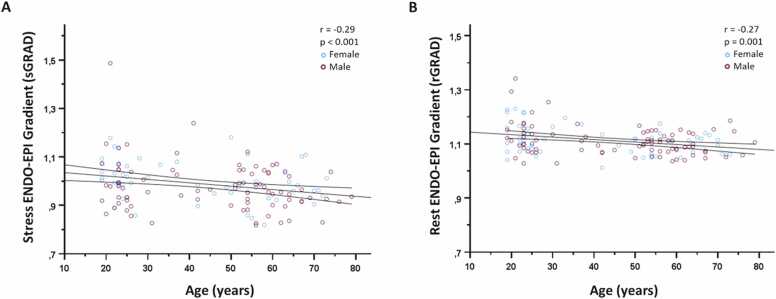
Correlation between (A) sGRAD and age; (B) rGRAD and age. There is a significant inverse correlation between sGRAD as well as rGRAD with increased age. *sGRAD* hyperemic endocardial to epicardial gradient, *rGRAD* rest endocardial to epicardial gradient

Fig. 3 A and B show a significant inverse correlation between sGRAD (r = −0.29, p<0.001) and rGRAD (r = −0.27, p = 0.001) values with increasing age.

### 7.3. Slice-specific values

Table 6 gives the slice-specific rest and stress endo- to epicardial gradient values for the entire cohort, and separately for female and male participants. There was no significant gender difference of endocardial to epicardial gradient between basal, mid and apical slices, except for the apical rest apical slices, which were significantly higher in female in comparison to male (p<0.001).

**Table 6:** Myocardial slice-specific ENDO to EPI gradient.

Parameters	All (n = 138)	Female (n = 54)	Male (n = 84)	p-value (F:M)
sGRAD				
Basal slice	0.98 ± 0.19	1.00 ± 0.21	0.95 ± 0.16	0.007
Mid slice	0.92 ± 0.11	0.91 ± 0.08	0.92 ± 0.12	0.436
Apical slice	1.03 ± 0.22	1.10 ± 0.21	1.01 ± 0.16	0.059
rGRAD				
Basal slice	1.08 ± 0.09	1.08 ± 0.09	1.08 ± 0.09	0.948
Mid slice	1.09 ± 0.07	1.08 ± 0.05	1.09 ± 0.08	0.303
Apical slice	1.16 ± 0.12	1.20 ± 0.11	1.14 ± 0.10	0.001

Values are given as median (±IQR) or mean (±SD). The p-value indicates the significance of differences between sex. *ENDO* endocardial layer, *EPI* epicardial layer, *sGRAD* hyperemic endocardial to epicardial gradient, *rGRAD* rest endocardial to epicardial gradient, *IQR* interquartile range, *SD* standard deviation

In the entire cohort, mid slice sGRAD was significantly lower in comparison to basal sGRAD (0.92 ± 0.11 vs. 0.98 ± 0.19; p<0.001) and apical slice sGRAD (0.92 ± 0.11 vs. 1.03 ± 0.22; p<0.001). Basal slice sGRAD was significantly lower in comparison to apical slice sGRAD (0.98 ± 0.19 vs. 1.03 ± 0.22; p = 0.007).

Mid slice rGRAD and basal slice rGRAD were significantly lower in comparison to apical rGRAD (1.09 ± 0.07 vs. 1.16 ± 0.12; p<0.001 and 1.08 ± 0.09 vs. 1.16 ± 0.12; p<0.001). There was no significant difference between mid slice rGRAD and basal slice rGRAD (1.09 ± 0.07 vs. 1.08 ± 0.09; p = 0.628).

## 8. Slice-specific normal values

Table 7 shows slice-specific normal values for MPR_ENDO_, MPR_EPI_, sGRAD and rGRAD as Median ± IQR values and [95% confidence interval].

**Table 7:** Normal values for slice-specific MPR_ENDO_, MPR_EPI_, sGRAD, and rGRAD.

Parameters	Basal slices	Mid slices	Apical slices
*MPR_ENDO_*			
Median±IQR [95% CI]	3.52 ± 1.32 [3.35–3.62]	3.12 ± 1.09 [2.96–3.33]	3.42 ± 1.34 [3.23–3.61]
*MPR_EPI_*			
Median±IQR [95% CI]	3.99 ± 1.27 [3.73–4.12]	3.77 ± 1.30 [3.53–3.93]	3.83 ± 1.43 [3.61–4.05]
*sGRAD*			
Median±IQR [95% CI]	0.98 ± 0.13 [0.95–1.00]	0.92 ± 0.11 [0.90–0.94]	1.03 ± 0.22 [1.00–1.06]
*rGRAD*			
Median±IQR [95% CI]	1.08 ± 0.07 [1.07–1.10]	1.09 ± 0.07 [1.07–1.10]	1.16 ± 0.12 [1.14–1.17]

*CI* confidence interval, *IQR* interquartile range, *MPR_ENDO_* myocardial perfusion reserve in the endocardial layer, *MPR_EPI_* Myocardial perfusion reserve in the epicardial layer, *sGRAD* hyperemic endocardial to epicardial gradient, *rGRAD* rest endocardial to epicardial gradient

Values are given as median ±IQR

## 9. Discussion

To the best of our knowledge, this is the first report of normal values of MPR_ENDO_, MPR_EPI_, sGRAD and rGRAD using automated in-line automated MBF quantification by CMR. We report both global and slice-specific values, to account for differences in how the three slices were acquired. Importantly, the reported values are specific to the pulse sequence, acquisition timing of each slice and analysis method used in this work and may differ for other methods. The study included a large cohort of healthy male and female volunteers from two separate centers and with a wide age range. Our results demonstrate a significant difference of the subendocardial and subepicardial perfusion values between the basal, mid and apical slices, as well as a significant reduction in these perfusion metrics with increasing age.

### 9.1. Dynamics of ENDO/EPI myocardial perfusion

Previous studies have investigated the dynamics of blood flow in the subepicardial and subendocardial layer under stress and rest conditions using radio-labeled microspheres [22]. Our observations are consistent with these previous studies, showing that rMBF_ENDO_ was significantly higher than rMBF_EPI_. This relationship is inverted under stress conditions, with sMBF_ENDO_ being significantly lower than sMBF_EPI_. As a consequence, the endo-epi gradient of MBF was significantly lower at stress (sGRAD) than at rest (rGRAD) and the MBF reserve in the endocardium (MPR_ENDO_) was significantly lower than the MBF reserve in the epicardium (MPR_EPI_). The primary factor believed to underlie these MBF disparities between the endocardium and epicardium is the compressive force exerted by the LV blood pool on the endocardium [23]. In response, coronary autoregulatory mechanisms induce a reduction in endocardial coronary vascular tone and an increase in the rMBF_ENDO_ in comparison to the rMBF_EPI_. Similarly, the reduced sMBF_ENDO_ in comparison to sMBF_EPI_ could also result from the compressive force of the LV blood pool on the endocardium [23]. Overall, these effects also lead to a reduction in MPR_ENDO_ in comparison to MPR_EPI_, as observed in this study.

### 9.2. Slice-specific ENDO/EPI perfusion metrics

We observed significant differences in transmural perfusion metrics between myocardial slices (Table 4, Table 6). This observation is likely influenced by the sequential acquisition of the myocardial slices across the R-R interval that is typical for 2D myocardial perfusion CMR (in our protocol in the order basal-mid-apical), with the consequence that each slice is acquired in a different cardiac phase (end-diastole/systole/early diastole). In our study, the mid-LV slice, which is acquired in systole, had lower perfusion metrics compared to the basal and apical LV slices, which were acquired during diastole. These differences in slice-derived parameters rGRAD and sGRAD align with previous findings [24], suggesting either varied transmural perfusion gradients across different LV locations or cardiac phases, for example due to higher compressive forces in systole, particularly affecting the endocardial layer, or that partial volume effects may affect thinner (diastolic) slices more than thicker (systolic) in relation with the limited in-plane acquisition resolution. The physiological tapering of wall thickness from base to apex, along with the conical shape of the LV apex, makes the apical slice particularly susceptible to partial volume effects. We therefore suggest that for 2D myocardial perfusion CMR, the mid slice myocardial perfusion metrics (assuming this slice is acquired at systole) is more accurate than basal and apical slices.

### 9.3. Age and ENDO/EPI myocardial perfusion

The observed age-related disparities in transmural perfusion metrics may be attributed to the global aging process of the cardiovascular system [25]. The suspected contribution of increased resting workload in the age-related inverse correlation of MPR_ENDO_ and MPR_EPI_ may be confirmed by the absence of significant age-related inverse correlation after RPP adjustment. We also observed an age-related inverse reduction of sMBF_ENDO_ and sMBF_EPI_ in absence of any significant age-related correlation with rest transmural perfusion values. This observation aligns with a recent publication on sMBF [26], and similar findings were reported for sMBF in participants over 70 y of age [27]. Our observation implies that the aging process results in a reduction of sMBF_ENDO_ and sMBF_EPI_ rather than rMBF_ENDO_ and rMBF_EPI_. In a recent CMR study of healthy volunteers with age distribution similar to our study, increased ventricular stiffness was observed with increasing age [28]. This age-related myocardial remodeling results in thickening of myocardial arterioles and alterations in microvascular vasomotion, further explaining the decline of sMBF_ENDO_ and sMBF_EPI_ with increasing age, as observed in our study [25].

### 9.4. Sex and ENDO/EPI myocardial perfusion

Prior investigations have consistently reported sex-specific disparities in myocardial perfusion parameters during stress and at rest, irrespective of the imaging techniques used [14], [29]. In our study, we have demonstrated that these differences affect both myocardial layers, with significantly higher absolute endocardial and epicardial MBF at rest and under stress in females compared to males (Table 3). The pathophysiological mechanisms underpinning these observations remain unclear. One potential explanation for the observed disparities in absolute MBF is the difference in extracellular volume frac (ECV) between females and males, as shown in previous research [30]. As postulated by the authors [30], higher ECV in females in comparison to males with the resulting increased gap between cardiomyocytes may explain their increased requirement for resting perfusion. Furthermore, the authors [30] postulated that higher MBF values during stress, on the other hand, may be explained by the higher capillary density observed in females. Moreover, there may be a bias when comparing female with male endo-epi gradients, since ENDO MBF may be more prone to partial volume effects in thinner myocardium, as previously discussed. Further research is warranted to comprehensively unravel the intricate factors contributing to these sex-specific variations in myocardial perfusion parameters.

### 9.5. Comparison with previous studies of transmural myocardial perfusion

Only small previous studies have reported on transmural perfusion metrics using quantitative myocardial perfusion CMR [31], [32]. In a study by Muehling et al. [31] involving 17 healthy volunteers, with a mean age of 34 ± 9 y, the investigators reported mean MPR_ENDO_ of 3.5 ± 1.4 and mean MPR_EPI_ of 4.8 ± 1.8, which was comparable to the corresponding age group in our study. Similar to our study, mean MPR_EPI_ was higher than mean MPR_ENDO_. In a study by Larghat et al. [32] with 17 healthy volunteers (mean age 34 ± 8 y), the investigators reported mean sGRAD of 0.91 ± 0.11 and mean rGRAD of 1.17 ± 0.16, similar to those obtain in our present study. However, the mean MPR_ENDO_ and MPR_EPI_ in [32] were lower than those obtained in our present study (2.6 ± 0.75 vs 3.3 ± 0.8 and 3.32 ± 0.93 vs 3.8 ± 0.9). The quantitative perfusion method in this previous study differed to ours and may in part account for the different results. Indeed, the authors [32] used only a single myocardial slice that was optimized for spatial resolution, contrast and motion for the image acquisition, which differs from our current acquisition protocol. Moreover, we used fully automated data processing with higher reproducibility [33], which differs from the off-line manual data processing in that work. In a study by Fairbairn et al. [34] that included rest, cold pressor and adenosine stress perfusion CMR in 19 healthy non-smoking participants (mean age 22 ± 6 y), estimates of sGRAD during adenosine stress and rGRAD were 0.89 and 1.1, similar to those obtained from mid myocardial slice transmural perfusion metrics in our cohort (Table 7). Using Rubidium-82 [^82^Rb] PET/CT, Gould et al. reported reference values for endocardial metrics obtained in a population of 120 healthy volunteers aged up to 40 y [12]. The normal mean sGRAD and MPR_ENDO_ were 0.94 ± 0.12 and. 1.02 ± 0.11. While the sGRAD value was similar to the mid myocardial slice transmural perfusion metrics of our study, the MPR_ENDO_ by PET/CT was surprisingly low, even if compared to whole myocardial values [35]. However, perfusion metrics given in this publication [12] were ratios obtained from relative images, and not from quantitative perfusion metrics expressed in mL/min/g. Moreover, the reduced spatial resolution of PET/CT in comparison to CMR could have contributed to underestimation of the transmural perfusion metrics due to partial volume effect.

### 9.6. Clinical implications

In-line automated pixel-wise MBF quantification [7] has become more widely available with over 100 centers worldwide having adopted this new methodology (dual-bolus in-line automated MBF estimation using the Gadgetron process. Thus, establishing normal values based on a large healthy population will help to implement these perfusion metrics in daily clinical practice. Previous investigations showed that the presence of focal or diffuse CAD leads to decreased intracoronary perfusion pressure during hyperemic stress. This results in reduced endocardial perfusion as well as sGRAD [12], [36], [37]. This adaptative sGRAD reduction is only possible if the integrity of the microvasculature and its vasodilatory adaptation are preserved [12], [38]. This physiological principle is fundamental for the correct differentiation between focal or diffuse CAD and MVD as the leading cause for diffuse reduction of sMBF and MPR, as shown for PET/CT [12], [38]. Using high-resolution CMR and dual-bolus acquisition sequence, Rahman et al. demonstrated in a population with angina that MPR_ENDO_ as well as sGRAD are useful parameters for the diagnostic of MVD, with MPR_ENDO_ having the highest accuracy for the diagnostic of MVD [1]. Furthermore, Chiribiri et al. demonstrated the accuracy of transmural perfusion gradient on high-resolution CMR to determine hemodynamically significant CAD as defined by fractional flow reserve [4]. In this setting, quantitative MBF assessment across the whole myocardium would be limited in the differentiation between diffuse CAD and MVD and the transmural indices reported here may aid to further identify these both pathologies [38]. Another potential clinical application of the described perfusion metrics could be in post-cardiac transplantation patients for the assessment of cardiac allograft vasculopathy. Muehling et al. have previously demonstrated that rest ENDO to EPI ratio could help to diagnose cardiac allograft vasculopathy [39].

## 10. Limitations

Compliance of the participants to refrain from caffeine-containing food or beverage 24 h prior to the scan was not confirmed by measuring caffeine levels. Previous evidence from a large cohort showed 5% prevalence of caffeine detection in patients undergoing non-invasive MPI [40]. Nevertheless, based on the monitoring of haemodynamic parameters during the stress test, we observed the expected physiological response in the presence of an adequate adenosine stress test. The absence of CAD in our cohort was based on clinical assessment and the exclusion of any subjects displaying regional perfusion defects on visual CMR assessment rather than coronary imaging, which would have been unethical to perform in this healthy cohort. While we made every effort to recruit healthy volunteers for this study, it cannot be excluded that some had an unknown disease that affected the findings. This study was conducted on MRI scanners from a single vendor (Siemens) at one field strength (3T), used a single stress agent (adenosine), and only one of several proposed quantitative myocardial perfusion CMR methods. Therefore, our results, in particular the absolute MBF values, may not be directly applicable to other acquisition and post-processing methods. However, many of the perfusion metrics in this study are ratios, making them more transferable to other acquisition and post-processing methods, as reported by Abraham et al. [41]. With a nonlinear change of MBF across the LV wall there may be other metrics that better capture the variation of MBF across the wall, such as the profiles of the transmural MBF variation. These may be subject of future investigations.

## 11. Conclusion

We propose global, slice-specific normal values of MPR_ENDO_, MPR_EPI_, sGRAD and rGRAD using automated in-line automated MBF quantification by CMR. These normal values may improve the MBF assessment in patients with suspected focal or diffuse CAD as well as MVD.

## Funding

CHK is supported by the development fund of the Lausanne University Teaching Hospital. SP is supported by 10.13039/501100000274British Heart Foundation CH/16/2/32089 and in part by the National Institute for Health and Care Research (NIHR) Leeds Biomedical Research Centre (BRC) (NIHR203331). The views expressed are those of the author(s) and not necessarily those of the NHS, the NIHR or the Department of Health and Social Care. GPM is supported by a NIHR Research Professorship (2017–08-ST2–007) and the NIHR Leicester Biomedical Research Centre. EL is supported by Wellcome Trust Clinical Career Development Fellowship (grant number: 221690/Z/20/Z) and is supported in part by the National Institute for Health and Care Research (NIHR) Leeds Biomedical Research Centre (BRC) (NIHR203331). The views expressed are those of the author(s) and not necessarily those of the NHS, the NIHR or the Department of Health and Social Care. RT is supported by a Romanian Society of Cardiology Research Grant (contract no 350/14.09.2023).

## Author contributions

**Christel H. Kamani:** Writing – review & editing, Writing –original draft, Methodology, Investigation, Formal analysis, Data curation,Conceptualization. **Louise Brown:** Writing – review & editing, Investigation, Data curation. **Thomas Anderton:** Writing – review & editing, Methodology. **Raluca Tomoaia:** Writing – review & editing, Methodology. **Chin Soo:** Writing – review & editing, Methodology. **Gaurav S. Gulsin:** Writing – review & editing, Methodology. **David A. Broadbent:** Writing – review & editing, Methodology. **Hui Xue:** Writing – review & editing, Methodology. **Jian L. Yeo:** Writing – review & editing, Methodology. **Alice L. Wood:** Writing – review & editing, Methodology. **Christopher E.D. Saunderson:** Writing – review & editing, Methodology. **Ioannis Botis:** Writing – review & editing, Methodology. **Arka Das:** Writing – review & editing, Methodology. **Nicholas Jex:** Writing – review & editing, Methodology. **Amrit Chowdhary:** Writing – review & editing, Methodology. **Sharmaine Thirunavukarasu:** Writing – review & editing, Methodology. **Noor Sharrack:** Writing – review & editing, Methodology. **Peter P. Swoboda:** Writing – review & editing, Methodology. **Hui Xue:** Writing – review & editing, Methodology. **John P. Greenwood:** Writing – review & editing, Methodology. **David Adlam:** Writing – review & editing, Methodology. **Eylem Levelt:** Writing – review & editing, Methodology. **Gerry P. McCann:** Writing – review & editing, Methodology. **Peter Kellman:** Writing – review & editing, Methodology. **Sven Plein:** Writing – review & editing, Validation, Supervision, Methodology, Conceptualization.

## Declaration of competing interests

The authors declare the following financial interests/personal relationships which may be considered as potential competing interests: Gerry McCann reports that financial support was provided by NIHR Leicester Biomedical Research Centre. Eylem Levelt reports that financial support was provided by Leeds Biomedical Research Centre. Raluca Tomoaia reports that financial support was provided by Romanian Society of cardiology. Christel Hermann Kamani reports that financial support was provided by Lausanne University Hospital. John Greenwood reports a relationship with Bayer AG that includes consulting or advisory. Eylem Levelt reports a relationship with Bristol Myers Squibb Co that includes consulting or advisory. Other authors declare that they have no known competing financial interests or personal relationships that could have appeared to influence the work reported in this paper.

## Data Availability

The datasets generated during and/or analyzed during the current study are available from the corresponding author on reasonable request.
