# Supplementary material for: Normal values of high-resolution transmural perfusion distribution metrics for automated quantitative pixel-wise myocardial perfusion cardiovascular magnetic resonance
Source: J Cardiovasc Magn Reson. 2025 Jun 19;27(2):101927. doi: 10.1016/j.jocmr.2025.101927 (PMC12445411; doi:10.1016/j.jocmr.2025.101927)
Supplement: Supplementary file 1 — Supplementary material [file mmc1.docx]

**Appendix**

| **Supplementary Table 1: Age- and sex-matched endo-epicardial perfusion metrics according to recruiting centre** | | | |
| --- | --- | --- | --- |
| **Parameters** | **Leeds (n=13)** | **Leicester (n=13)** | **p-value** |
| MPR_ENDO_ | 3.6 ± 1.0 | 3.3 ± 0.9 | 0.40 |
| MPR_EPI_ | 4.1 ± 1.1 | 3.7 ± 1.1 | 0.38 |
| sGRAD | 0.98 ± 0.09 | 0.97 ± 0.07 | 0.74 |
| rGRAD | 1.10 ± 0.03 | 1.07 ± 0.03 | 0.05 |
| rGRADc | 1.10 ± 0.03 | 1.08 ± 0.04 | 0.13 |
| *Values are given as mean (±SD). The p-value indicates the significance of differences between the two investigations sites (Leeds and Leicester).* | | | |

| **Supplementary Table 2: Age- and sex-matched endo-epicardial perfusion metrics according to contrast agent dose** | | | |
| --- | --- | --- | --- |
| **Parameters** | **Gd: 0.05 mmol/kg (n=10)** | **Gd: 0.075 mmol/kg**  **(n=10)** | **p-value** |
| MPR_ENDO_ | 3.5 ± 0.9 | 3.0 ± 0.6 | 0.20 |
| MPR_EPI_ | 3.8 ± 0.9 | 3.5 ± 0.8 | 0.40 |
| sGRAD | 1.03 ± 0.11 | 0.95 ± 0.08 | 0.08 |
| rGRAD | 1.11 ± 0.03 | 1.08 ± 0.04 | 0.04 |
| rGRADc | 1.11 ± 0.07 | 1.07 ± 0.07 | 0.07 |
| *Values are given as mean (±SD). The p-value indicates the significance of differences between different intravenous contract agent dosage (0.075 mmol/kg versus 0.05 mmol/kg gadolinium).*  *Gd: gadolinium; mmol/kg: millimole per kilogram.* | | | |

| **Supplementary Table 3: Regional endocardial and epicardial sMBF, rMBF and MPR** | | | | |
| --- | --- | --- | --- | --- |
| **Parameters** | **All**  **n=138** | **Female**  **n=54** | **Male**  **n=84** | **p-value**  **(M:F)** |
| rMBF_ENDO-LAD_ | 0.68 ± 0.19 | 0.76 ± 0.14 | 0.64 ± 0.18 | <0.001 |
| rMBF_ENDO-LCX_ | 0.62 ± 0.18 | 0.70 ± 0.14 | 0.58 ± 0.17 | <0.001 |
| rMBF_ENDO-RCA_ | 0.61 ± 0.12 | 0.66 ± 0.11 | 0.58 ± 0.11 | <0.001 |
| rMBF_EPI-LAD_ | 0.62 ± 0.19 | 0.70 ± 0.14 | 0.59 ± 0.18 | <0.001 |
| rMBF_EPI-LCX_ | 0.52 ± 0.18 | 0.59 ± 0.14 | 0.51 ± 0.17 | <0.001 |
| rMBF_EPI-RCA_ | 0.56 ± 0.12 | 0.61 ± 0.11 | 0.53 ± 0.11 | <0.001 |
| sMBF_ENDO-LAD_ | 2.32 ± 0.55 | 2.51 ± 0.51 | 2.21 ± 0.54 | <0.001 |
| sMBF_ENDO-LCX_ | 2.17 ± 0.56 | 2.39 ± 0.54 | 2.04 ± 0.54 | <0.001 |
| sMBF_ENDO-RCA_ | 2.09 ± 0.49 | 2.24 ± 0.48 | 2.00 ± 0.47 | 0.002 |
| sMBF_EPI-LAD_ | 2.35 ± 0.64 | 2.53 ± 0.54 | 2.21 ± 0.61 | 0.003 |
| sMBF_EPI-LCX_ | 2.26 ± 0.58 | 2.40 ± 0.53 | 2.17 ± 0.59 | 0.012 |
| sMBF_EPI-RCA_ | 2.10 ± 0.52 | 2.21 ± 0.50 | 2.04 ± 0.52 | 0.042 |
| MPR_ENDO-LAD_ | 3.4 ± 0.9 | 3.4 ± 0.9 | 3.5 ± 0.9 | 0.797 |
| MPR_ENDO-LCX_ | 3.4 ± 1.5 | 3.5 ± 1.1 | 3.4 ± 1.5 | 0.886 |
| MPR_ENDO-RCA_ | 3.4 ± 1.2 | 3.5 ± 0.9 | 3.5 ± 1.2 | 0.720 |
| MPR_EPI-LAD_ | 3.6 ± 1.2 | 3.7 ± 1.1 | 3.9 ± 1.0 | 0.380 |
| MPR_EPI-LCX_ | 4.3 ± 1.3 | 4.3 ± 1.9 | 4.4 ± 1.3 | 0.726 |
| MPR_EPI-RCA_ | 3.7 ± 1.1 | 3.6 ± 1.2 | 3.8 ± 1.1 | 0.227 |
| *Values are given as median (±IQR) or mean (±SD). The p-value indicates the significance of differences between sex.* | | | | |

| **Supplementary Table 4: Slice-specific endocardial and epicardial sMBF and rMBF** | | | | |
| --- | --- | --- | --- | --- |
| **Parameters** | **All**  **n=138** | **Female**  **n=54** | **Male**  **n=84** | **p-value**  **(M:F)** |
| **rMBF_ENDO_** |  |  |  |  |
| Basal slice | 0.65 ± 0.18 | 0.71 ± 0.13 | 0.61 ± 0.18 | <0.001 |
| Mid slice | 0.64 ± 0.19 | 0.72 ± 0.13 | 0.60 ± 0.16 | <0.001 |
| Apical slice | 0.62 ± 0.19 | 0.69 ± 0.14 | 0.56 ± 0.17 | <0.001 |
| **rMBF_EPI_** |  |  |  |  |
| Basal slice | 0.59 ± 0.19 | 0.65 ± 0.13 | 0.55 ± 0.18 | <0.001 |
| Mid slice | 0.58 ± 0.18 | 0.67 ± 0.13 | 0.54 ± 0.15 | <0.001 |
| Apical slice | 0.52 ± 0.17 | 0.58 ± 0.13 | 0.50 ± 0.14 | <0.001 |
| **sMBF_ENDO_** |  |  |  |  |
| Basal slice | 2.32 ± 0.88 | 2.60 ± 0.62 | 2.20 ± 0.60 | <0.001 |
| Mid slice | 2.08 ± 0.48 | 2.22 ± 0.47 | 1.99 ± 0.47 | 0.002 |
| Apical slice | 2.17 ± 0.53 | 2.32 ± 0.54 | 2.08 ± 0.50 | 0.006 |
| **sMBF_EPI_** |  |  |  |  |
| Basal slice | 2.32 ± 0.74 | 2.50 ± 0.57 | 2.20 ± 0.73 | 0.008 |
| Mid slice | 2.24 ± 0.65 | 2.44 ± 0.52 | 2.16 ± 0.56 | <0.001 |
| Apical slice | 2.06 ± 0.63 | 2.14 ± 0.49 | 2.02 ± 0.58 | 0.162 |
| *Values are given as median (±IQR) or mean (±SD). The p-value indicates the significance of differences between sex.* | | | | |
